# Telehealth Visits After Shoulder Surgery: Higher Patient Satisfaction and Lower Costs

**DOI:** 10.5435/JAAOSGlobal-D-22-00119

**Published:** 2022-07-06

**Authors:** Evan A. O'Donnell, Jillian E. Haberli, Andres Muniz Martinez, Daniel Yagoda, Robert S. Kaplan, Jon J. P. Warner

**Affiliations:** From the Department of Orthopaedic Surgery, Massachusetts General Hospital, Boston, MA (Dr. O'Donnell, Haberli, Dr. Martinez, and Dr. Warner); the Avant-garde Health, Boston, MA (Yagoda); and the Harvard Business School, Boston, MA (Dr. Kaplan).

## Abstract

**Introduction:**

Studies comparing the cost of in-person and virtual care are lacking. The goal of this study was threefold (1) to compare the cost of telemedicine visits with in-person clinic visits after common shoulder surgeries, (2) to measure the safety, and (3) to evaluate patient experience with telemedicine visits.

**Methods:**

The In-Person Visit cohort (N = 25) and the telemedicine cohort (Virtual Visit cohort, N = 24) were selected from patients undergoing routine follow-up of common shoulder procedures. Time-driven activity-based costing was used to determine costs associated with each episode of care. Patient complications, satisfaction, convenience, and technical difficulties associated with telehealth were recorded.

**Results:**

The average Virtual Visit was 54.1% less costly and 87.8% shorter than the In-Person Visit ($49 versus $107 per patient, 8.6 versus 70.1 minutes per patient, *P* < 0.01, respectively). One complication was missed in the Virtual Visit cohort, later captured by an in-person visit. All patients in the Virtual Visit cohort reported that the virtual visit was safe and convenient and showed high levels of satisfaction.

**Discussion:**

Virtual visits for postoperative care of patients undergoing shoulder surgery are associated with decreased costs and high ratings of convenience and satisfaction. Postoperative complications may be more challenging to diagnose virtually.

Physicians and surgeons across all specialties have taken part in a healthcare paradigm transformation toward the utilization of telemedicine. The adoption of telemedicine, the delivery of health care through a remote communication platform, has been fueled by the emergence of severe acute respiratory syndrome coronavirus 2 (SARS-CoV-2) and the associated respiratory disease coronavirus 2019 (COVID-19) pandemic. In a single year, the utilization of telemedicine in the United States grew from 15,000 interactions annually to over 24.5 million interactions annually, representing a 1,600-fold increase.^1^ Within the field of orthopaedic surgery, telemedicine has shown extensive promise in many phases of the patient's treatment cycle.^2,3,4,5^ Although telemedicine offers the potential to reduce costs and improve patient satisfaction, such benefits have yet to be quantified.

Practice is also shifting from an emphasis on volume to one of value, specifically value-based health care, which strives to improve patient outcomes while lowering the cost of care. To measure the cost component of value-based health care, clinicians are increasingly using time-driven activity-based costing (TDABC).^6^ TDABC involves the mapping of clinical processes and measuring the time that each clinical staff person and equipment type interacts with patients over a complete care cycle.^6^ The interactions are described by two parameters, the time spent by each personnel or equipment type during the care cycle and the capacity cost rate (CCR; cost per minute) of the aforementioned resources.^6^ When multiplied together and summed across all personnel and equipment, the method yields the total direct cost of care. TDABC has become the new cost accounting standard for orthopaedic research.^7,8,9,10^ Although several authors have reported on cost reductions with the use of telemedicine, to date there are no reports of cost comparison between in-person visits and telemedicine visits in orthopaedic care.

In this study, the goal of this study was to use the TDABC methodology (1) to analyze the cost of telemedicine virtual visits and in-person clinic visits for routine follow-up of common shoulder surgeries, (2) to determine the drivers of cost variation between each type of visit, and (3) to evaluate the patient experience including satisfaction and safety with telemedicine virtual visits.

## Methods

### Study Design

This study received institutional review board approval. It was designed with two groups of 25 patients: an in-person clinic-visit cohort (In-Person Visit cohort) and a telemedicine cohort (Virtual Visit cohort). The patients in both groups required a routine, postoperative visit after shoulder surgery. The In-Person Visit cohort presented for a 2-week follow-up, whereas the Virtual Visit cohort was seen 7 to 10 days after surgery and in addition had a 2-week in-person follow-up. This redundant visit was used as a safety control subject, to address concerns, if any, which arose and were unresolvable during the virtual visit. Patients in the Virtual Visit cohort were enrolled at their standard preoperative visit and were a consecutive cohort. The In-Person Visit cohort was a convenience cohort, the data were deidentified, and no consent was obtained.

### Virtual Visits

The telehealth visits were conducted using Zoom (Zoom Video Communications), which was a software embedded within the electronic medical record software Epic (Epic Systems). The visits were conducted with audio and visual interaction between patient and provider. A standard history and virtual physical examination were conducted. Because this represented acute postoperative care, physical examination was focused on incision inspection and assessment of neurovascular status by hand function and sensation.

### Time-driven Activity-based Costing

**Figure 1:**
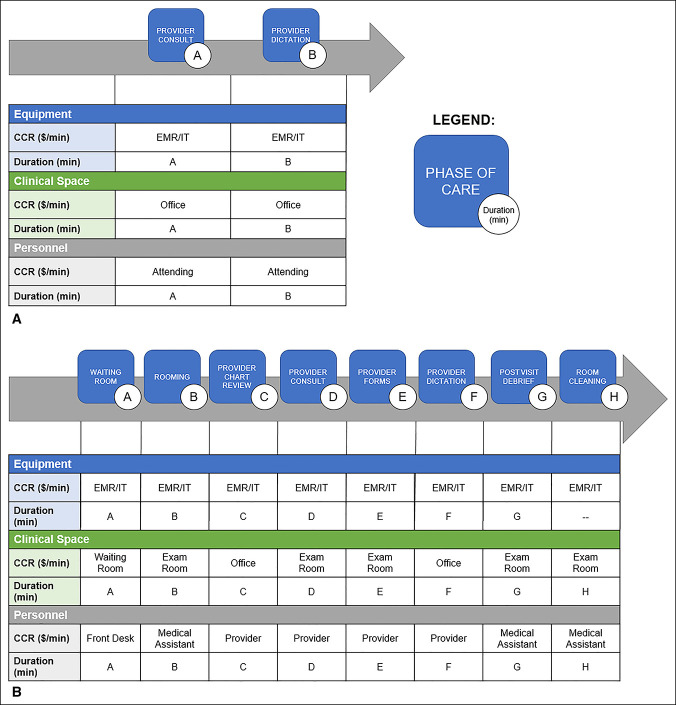
**A** and **B**, Diagrams showing process maps for TDABC. **A**, To account for cost, cost capacity rates (CCRs, $/min) were multiplied by duration (minutes) for each phase of care the patient entered (**A** and **B**). CCRs were established for equipment, clinical space, and personnel types. **A**, Duration of the provider consult and (**B**) duration of the provider dictation. EMR/IT: The CCR ($/min) for the use of the electronic medical record and associated information technology including hardware, software, updates, maintenance, etc. **B**, To account for cost, cost capacity rates (CCRs, $/min) were multiplied by duration (minutes) for each phase of care the patient entered (**A**–**H**). CCRs were established for equipment, clinical space, and personnel types. **A**, Duration in waiting room, (**B**) duration in rooming (minutes), etc. EMR/IT: The CCR ($/min) for use of the electronic medical record and associated information technology including hardware, software, updates, maintenance, etc.

TDABC provides accurate and granular cost attributed to a care episode. The calculation is a function of two parameters for each resource provided: CCR and the time duration the resource was used.^11^ To this end, process maps were developed for the In-Person Visit and Virtual Visit cohorts, which diagrammed the patient resource interaction (Figure 1, A and B).^6^

Three types of resources were used for the In-Person Visit cohort: equipment, clinical space, and personnel. For equipment, the annual cost of the electronic medical record, hardware, software, desktop computers, tablets, printers, scanners, applications, upgrades, information technology assistance, redesign, training, and ongoing network fees were estimated from published national averages per provider (HealthIT.gov). The annual equipment costs were then converted to CCRs by division of the annual costs by total usable minutes attributed to the equipment. The CCR for clinical space was defined by regional cost rates per square foot of Boston, MA (Commercial Café, Santa Barbara, CA), and the office spaces used for patient care were measured manually. Personnel CCRs were developed for front desk receptionists for registration and wait time in lobby; medical assistant rooming and interview; provider chart review, consultation, dictation, and postconsultation forms; and medical assistant postvisit debriefing and room cleaning. The personnel CCRs were defined in collaboration with Avant-garde Health (Boston, MA) and were determined from published national salary averages (Glassdoor, Mill Valley, CA).

For the Virtual Visit cohort, the resources used included equipment, clinical space, and personnel. Equipment was estimated as discussed earlier. Clinical space included the attending office during the virtual visit. The only personnel costs were for the consultation and dictation time of the attending provider. The CCRs of each resource were defined as mentioned earlier. The duration each patient interacted with a given resource was manually time-stamped and recorded. Cost was then determined based on the resource CCR and the duration of patient interaction.

### Safety

Postoperative complications and any aberrations from routine follow-up care for the Virtual Visit cohort were recorded.

### Virtual Visit Patient Experience

**Figure 2:**
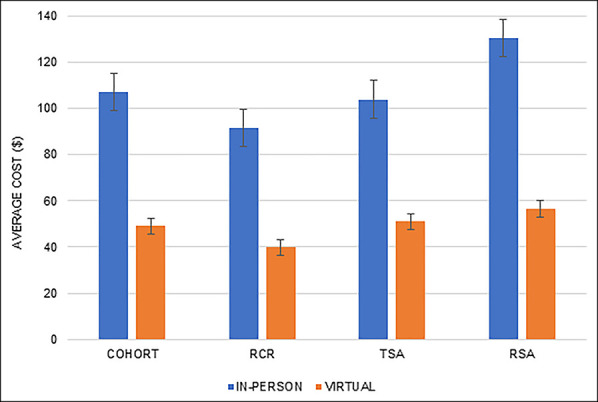
Graph showing average cost per visit.

The Virtual Visit cohort completed a survey to assess the patient experience (Figure 2, http://links.lww.com/JG9/A223). A Likert scale (1 to 5: strongly agree, agree, uncertain, disagree, and strongly disagree, respectively) was used to assess patient satisfaction, convenience, safety, telehealth connectivity difficulties, technology troubleshooting, and visit completeness. The survey also included the Net Promoter Score, a composite of patient experience and satisfaction, which asks the likelihood the patient would recommend virtual visits to a friend or colleague (0 to 10, extremely unlikely to extremely likely).^12^

### Statistical Methods

To ensure hospital, employee, and patient confidentiality, costs per unit time were derived from published national averages. The average cost per type of visit and phase of care were calculated and reported in $US. Chi square analysis compared the distribution of procedure type. Average cost and duration of virtual visits and in-person visits were compared with the Student two-tailed *t*-test. Statistical analyses were conducted in Microsoft Excel (Version 16.37). A *P* value of < 0.05 was considered statistically significant. The investigation did not receive external funding.

## Results

### Pooled Cohort

Fifty patients were enrolled in this study with only 2% attrition because one patient in the In-Person Visit cohort was excluded for incomplete timing data necessary for TDABC analysis. In sum, 24 patients composed the In-Person Visit cohort and 25 patients were in the Virtual Visit cohort. Among the 49 patients, 18 received rotator cuff repairs (RCRs, 36.7%), 17 underwent total shoulder arthroplasty (TSA, 34.7%), and 14 underwent reverse shoulder arthroplasty (RSA, 28.6%). The distribution of procedure types was not statistically different between the In-Person Visit cohort and Virtual Visit cohort (*P* = 0.13).

### TDABC Analysis

#### Cohort Comparison

The average Virtual Visit cost of $49 per patient was 54.1% less than the average In-Person Visit cost ($107 per patient, *P* < 0.01; Figure 2). The average Virtual Visit cost was significantly lower than the average In-Person Visit cost in RCR, TSA, and RSA subgroup analyses (*P* < 0.01, *P* = 0.02, and *P* < 0.01, respectively).

**Figure 3:**
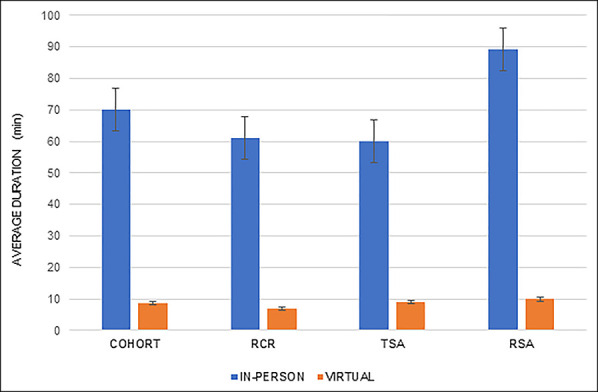
Graph showing average duration per visit

The average Virtual Visit episode of care duration was 8.6 minutes per patient, 88% less than the In-Person Visit episode of care (70.1 minutes per patient, *P* < 0.01; Figure 3). The average duration of RCR, TSA, and RSA subgroups was significantly shorter in the Virtual Visit cohort than the In-Person Visit duration (*P* < 0.01, *P* = 0.01, and *P* < 0.01, respectively). The patient-specific activity duration and associated costs are displayed in the Appendix, http://links.lww.com/JG9/A221 and http://links.lww.com/JG9/A222.

#### In-person Visit Cohort

**Figure 4:**
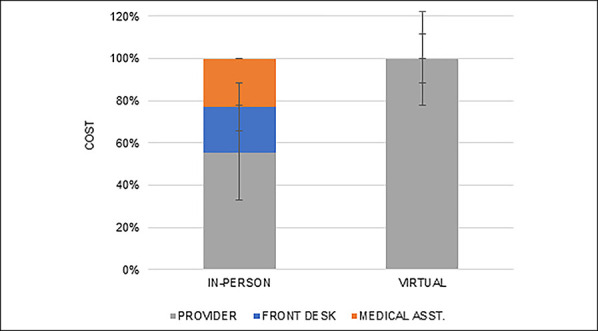
Graph showing in-person visit cost by personnel type (%)

Regarding the In-Person Visit cohort, the provider consultation accounted for the greatest cost per patient ($43 per patient, 39.8% of total episode cost), followed by waiting room cost ($23 per patient, 21.8%) and rooming cost ($21 per patient, 20.0%). Provider-associated activities accounted for 55.3% of cost per patient, and the medical assistant-associated and front desk-associated activities accounted for 22.8% and 21.8% of the cost per patient, respectively (Figure 4).

#### Virtual Visit Cohort

In the Virtual Visit cohort, the consultation time of the provider accounted for the greatest cost per patient ($41 per patient), 83.5% of the total episode cost per patient. Provider dictation accounted for $8 per patient and 16.5% of the total cost per patient. Provider-associated activities accounted for 100% of the associated cost per patient.

### Equipment, Clinical Space, and Personnel Cost Comparison

**Figure 5:**
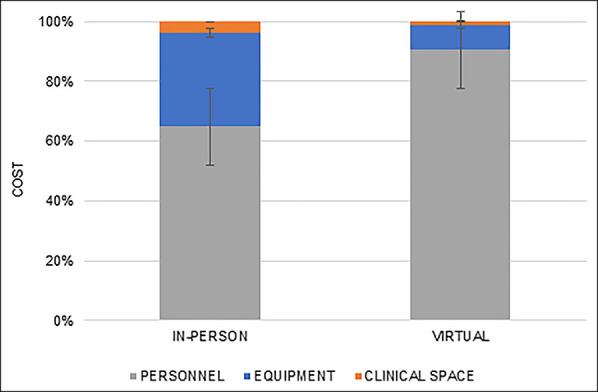
Graph showing in-person visit cost by personnel, equipment, and clinical space (%)

In both the Virtual Visit and In-Person cohorts, personnel accounted for the highest proportion of cost at 90.5% and 64.9% of the total cost, respectively (Figure 5). Equipment-associated costs were the second-most costly (8.4% versus 31.4%), and clinical space was the least costly (1.1% versus 3.8%) for the Virtual Visit and In-Person cohorts, respectively. The distribution of cost between equipment, clinical space, and personnel was significantly different between the Virtual Visit and In-Person cohorts (*P* < 0.01, Figure 5).

### Safety

There were no instances of a change in follow-up care within the Virtual Visit cohort. There was one complication noted within the Virtual Visit cohort, an incomplete axillary and suprascapular nerve palsy, presenting with incomplete loss of sensation to the deltoid patch. This complication was diagnosed at the 2-week follow-up visit and not identified during the telehealth visit. The patient was treated expectantly, underwent electrodiagnostic evaluation at the 6-week follow-up visit confirming the diagnosis, and had spontaneous improvement.

### Virtual Visit Survey

**Figure 6:**
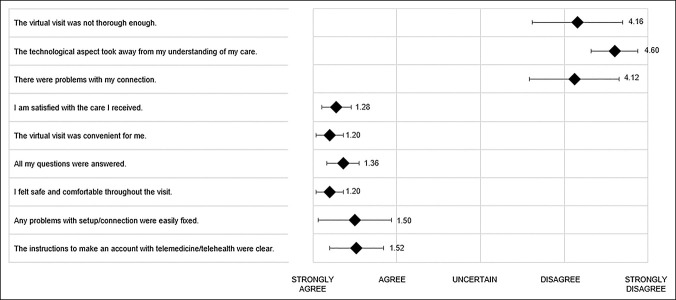
Diagram showing virtual visit survey data

All patients in the Virtual Visit cohort reported that the virtual visit was safe (1.2, SD 0.4) and convenient (1.2, SD 0.4) and showed high levels of satisfaction (1.3, SD 0.5) by the Likert scale (answering strongly agree or agree; Figure 6). Patients reported few difficulties with telehealth connection and that any technological problems were easily fixed (Figure 6). The average Net Promoter Score was 9.3 (of 10, extremely likely to recommend, SD 1.2).

## Discussion

This study described and compared the cost of in-person clinic visits and virtual visits, along with metrics on the patient experience and complications with telemedicine. Virtual visits cost 54% less than in-person visits and were convenient and high in patient satisfaction. One complication was missed by telehealth, ultimately diagnosed in-person, which did not require an immediate change in routine care. In summary, virtual visits offered an excellent patient experience at markedly less cost to the healthcare system, but continued research is warranted in defining which complications may be challenging to diagnose by telehealth.

Telemedicine has been used across all phases of orthopaedic surgery, from initial patient consultation^2,13^ to perioperative care^3-5^ and postoperative telerehabilitation.^14^ Reports on patient experience have generally been favorable. Shafi et al conducted telemedicine visits for both initial consultations and follow-up of spine disorders. The authors reported high levels of patient satisfaction (4.79/5), efficacy (4.32/5), and a high likelihood to recommend telemedicine to a friend (86.9%). Sharareh and Schwarzkopf^5^ reported on patients who underwent total joint arthroplasty and found that patients preferred telemedicine to in-person visits. Marsh et al also reported on total joint arthroplasty although noted only moderate-to-high patient satisfaction with virtual follow-up and higher satisfaction with in-person follow-up (92.8% versus 73.9%). The present study favors virtual follow-up, with high ratings of patient satisfaction, efficacy, and safety. Furthermore, using the Net Promoter Score, patients in the Virtual Visit cohort were extremely likely to recommend the experience (9.26/10).

Many authors have reported on the efficacy and patient satisfaction associated with telemedicine more broadly. Across all medical specialties, analyses of telemedicine in ophthalmology,^15^ psychiatry,^16^ cardiology,^17^ and intensivist programs^18^ have all demonstrated cost savings with the utilization of telemedicine. In addition, two systematic reviews of telemedicine and cost analyses have been reported, which both report favorably on the cost-efficacy of telemedicine.^19,20^

Few studies, however, have been conducted to calculate the cost savings from telemedicine. Harno et al and Ohinmaa et al reported on the cost reduction of telemedicine compared with in-person visits of orthopaedic patients and reported a 22.2% and 31.0% reduction in overall cost, respectively.^21,22^ These findings are consistent with our data, which showed a 54.1% cost reduction per patient with telemedicine. Furthermore, Harno et al^21^ reported that a notable driver of the cost savings from telemedicine visits was decreased cost of staffing, which is analogous to the findings of our study. The findings of the present study showed that personnel costs accounted for 64.9% of the in-person visits and 90.5% of the virtual visits. Although these cost analyses show favorable results for the use of telemedicine in orthopaedics, the authors used a heterogenous methodology in cost accounting that is much less accurate than TDABC analyses.

The cost savings from telemedicine in our study are driven by the vast reduction of visit duration. The average in-person visit episode of care was over 1 hour longer than the average virtual visit. This decrease likely results from the elimination or reduction in setup costs (eg, the time required to move patients between the office phases of care and the wait for medical professional interaction). Through telemedicine, the patients can be queued remotely, and the cost associated with other medical staff monitoring while the patient waits for the provider interaction is not incurred. In our study, the non–provider-associated costs accounted for 44.7% of the in-person visit cost, representing the likely setup cost. This cost may be eliminated in virtual visits, where nonproviders are not needed.

Although outside the scope of this study, the time saved by telehealth visits merits discussion beyond merely its cost-reducing benefit. By reducing the average episode of care duration by over 80%, capacity is increased markedly. Aside from patient care, where increased capacity could translate to higher volume of clinic visits or surgeries, surgeons and their staff contribute broadly to numerous initiatives within their departments, hospitals, and the healthcare system. By reducing the average patient visit duration and while maintaining high levels of patient satisfaction, our study suggests that telemedicine may offer the opportunity to redistribute orthopaedic staff time toward other value-creating activities.

Descriptions of safety and limitations of telehealth are scarce in orthopaedic literature. We report a single complication which was ultimately missed by telehealth visit and was later diagnosed during an in-person visit. It is important to note that neither the complication nor its management was adversely affected by the virtual health visit. Although the patient did not require any immediate intervention or change in their postoperative care, this does highlight the limitations of the physical examination and diagnostics when using telehealth. Moreover, with the introduction of a novel technology, there is an associated learning curve for optimization. Additional research is warranted in defining the weaknesses of telehealth and its application to orthopaedic care.

### Limitations

This study has several notable limitations. The methodology was not blinded, and as such, biases may persist between the in-person and virtual visit practices. The Virtual Visit cohort represents a consecutive cohort and may suffer selection bias. The patients who a priori felt uncomfortable with telehealth may have refused to participate in this study. This methodology may be selected for those patients who are more technologically savvy or for healthier and younger patients with limited disease burden which may complicate the virtual interfacing. This study did not analyze the time for routine imaging postoperatively, thus may not be generalizable for all routine follow-up care. The data represent the workflow of a single provider at a tertiary referral center for shoulder pathology and thus may not be generalizable to all subspecialties or practice settings. In particular, wait times contributed markedly to the overall episode-of-care cost in the In-Person cohort. A more efficient schedule may reduce these setup costs, although patient tardiness, physical limitations, and other characteristics will always ensure some wait time. The cost rates used to tabulate patient-specific costing were derived from publicly available national wage averages and not directly from employee data. Although this may change the absolute costs reported, the cost differences reported in this study would be preserved across comparisons of the in-person and virtual visits. Finally, there is no comparative analysis offered for patient satisfaction or perceived safety for in-person visits because in-person visits were thought to be the benchmark for care. Despite these limitations, this article serves as seminal evidence for both the cost-saving benefit of telemedicine in orthopaedic care and the limitations of in-physical examination and diagnostics.

### Conclusions

Virtual visits for routine postoperative care of patients undergoing shoulder surgery are associated with decreased costs and high ratings of convenience and satisfaction. Limitations in virtual physical examination and diagnostics warrant continued research into the application of this technology.
